# Polyethylene Terephthalate: Sax Responds

**DOI:** 10.1289/ehp.1001986R

**Published:** 2010-05

**Authors:** Leonard Sax

**Affiliations:** Montgomery Center for Research in Child & Adolescent Development, Exton, Pennsylvania, E-mail: mcrcad@verizon.net

In his letter Vasami reminds readers that phthalates are not used in the manufacture of PET. Indeed, I emphasized precisely this point in my commentary (Sax 2010), for example, when I stated that “phthalates are not used as substrates or precursors in the manufacture of PET.” Vasami also asserts that “it is not chemically plausible for PET to produce these phthalate esters”; however, I never suggested that virgin PET gives rise to phthalate esters via degradation of PET itself. I did cite multiple studies in which phthalates were recovered from the contents of PET bottles—as contaminants leaching from the PET bottle wall. How did the phthalates come to be there? As I noted, one possibility is that some of the PET used in manufacturing the bottles may have been recycled PET, and some of this recycled PET might have been contaminated with phthalates. Again, as I noted in my commentary, PET is commonly used for bottling a variety of products (e.g. shampoo) that are known to contain phthalates; these phthalates can then sorb into the PET bottle. Other researchers have previously documented that various organic substances readily migrate into PET (e.g., Komolprasert and Lawson 1997). Indeed, previous investigators have documented the presence of phthalates in PET bottles marketed for consumer use (e.g., Kim et al. 1990); Nerín et al. (2000) reported that the concentration of phthalates was much higher in recycled PET material than in virgin PET.

There are good environmental arguments for recycling plastics rather than disposing of them in landfills. The potential for tension between the desire to recycle plastics, on the one hand, and the desire to protect human health, on the other hand, has long been recognized (e.g., Castle 1994). Reconciling these two objectives requires a better understanding of the origin of endocrine disruptors in PET.

Vasami notes that although Choe et al. (2003) reported antimony chloride as showing high estrogenicity, “antimony oxides—not antimony chloride—are used as catalysts in the manufacture of PET.” Although Vasami asserts that antimony oxides “are chemically and toxicologically distinct from antimony chlorides,” the toxicological literature does not provide strong support for this assertion. Antimony chloride, when combined with water, readily forms antimony oxide (National Research Council 2000), and both antimony chloride and antimony oxide ionize *in vivo.* Merski et al. (2008) reported that when animals were fed ground PET, antimony was recovered from their urine in a dose-dependent fashion. Toxicologically, what seems to matter is the antimony and its oxidation state [trivalent (III) or pentavalent (V)], not the anion (chloride or oxide). Antimony(III) is the ionization state in the antimony oxide used in the production of PET; the same ionization state (III) is found in antimony chloride. Using X-ray spectrometry, Martin et al. (2010) confirmed that the antimony in PET bottle walls is in fact trivalent antimony. The toxicological literature clearly establishes that trivalent antimony is far more toxic to humans than is pentavalent antimony (e.g., Chulay et al. 1988; De Boeck et al. 2003; Phillips and Stanley 2006). Vasami’s implication that antimony(III) oxide, when ingested, might be free of the risks demonstrated for antimony(III) chloride, is without evidentiary basis.

Vasami concludes by reminding readers that PET bottles meet all applicable safety requirements. However, he neglects to note that these safety requirements were developed largely in the 1980s and 1990s, when the chief concern about antimony and other metalloids had to do with carcinogenicity (e.g., De Boeck et al. 2003) and organ toxicity (e.g., Poon et al. 1998). The standards were developed based on doses believed to be carcinogenic and/or directly toxic. The ability of inorganic metalloids such as antimony to act as xenoestrogens has only recently been recognized (Darbre 2006). More research is needed to determine whether the regulatory requirements for antimony in foods and beverages should be adjusted in order to minimize the risk of endocrine-disrupting effects.

Certainly there is a paucity of research on the endocrine-disrupting effects of antimony. But surely the remedy for this deficiency is more research, not a stubborn insistence that what we don’t know can’t hurt us.
